# Healthcare Practitioners’ Views on Management Practices of Self-Harm in Older Adults: A Qualitative Study Conducted in Ireland

**DOI:** 10.1192/bjo.2024.197

**Published:** 2024-08-01

**Authors:** Caoimhe Lonergan, M. Isabela Troya

**Affiliations:** 1 Department of Psychiatry and Neurobehavioural Science, University College Cork, Cork, Ireland; 2 South Lee Mental Health Services, Cork University Hospital, Cork, Ireland; 3 National Suicide Research Foundation, University College Cork, Cork, Ireland; 4 School of Public Health, College of Medicine and Health, University College Cork, Cork, Ireland

## Abstract

**Aims:**

To explore the views of healthcare practitioners from diverse clinical settings on management practices when supporting older adults with self-harm behaviour.

**Methods:**

Semi-structured interviews were conducted with healthcare practitioners with previous experience supporting older adults who self-harm, including consultant psychiatrists, general practitioners, clinical psychologists, psychotherapists, clinical nurse specialists and social workers. Purposive sampling was used to recruit participants in the Republic of Ireland to ensure a varied representation of location and clinical area. Healthcare practitioners were recruited by advertising the study via professional and clinical research networks, social media and snowballing methods. Interviews were audio-recorded and transcribed verbatim. Transcripts were uploaded to QSR NVivo Software Version 12 to facilitate analysis. Themes were identified in the data using the steps of thematic analysis which involve data familiarization, coding, theme development and revision.

**Results:**

Interviews were conducted with 20 healthcare practitioners from April to July 2023. Healthcare practitioners offered diverse perspectives across general practice, community mental health services, liaison psychiatry, emergency department settings and inpatient mental health units. Three main themes were generated:

1. Supporting older adults after self-harm: complex and challenging.
2. Multiple barriers to the management of self-harm: i) strained resources and unclear referral pathways, ii) limited awareness/health promotion, iii) unsuitable environments, iv) stigma and shame, and v) complexity of self-harm.
3. Risk assessment in older adults: increased risk and the importance of safety planning.

Relevant quotes from participants are provided to support these themes.

**Conclusion:**

Healthcare practitioners viewed self-harm in older adults as complex, challenging and associated with high suicide risk. Increased mental health promotion and awareness of mental health and suicidal behaviour in this age group would help address current stigma and shame. Primary care was identified as a sector that older adults often access and where prevention, identification and support can be offered, with more complex cases being promptly referred to more specialist services. Several supports and therapies that could help older adults were identified; however, due to the limited availability of services, supports were often restricted due to cut-off age criteria or disparity of care at a national level. Provision of care needs to be improved upon, with standardised supports still needing to be implemented across the country. Future research can address the perspectives of older adults on how they would prefer to be supported for their self-harm.

